# Semen Biochemical Components in Varicocele, Leukocytospermia, and Idiopathic Infertility

**DOI:** 10.1007/s43032-020-00260-0

**Published:** 2020-07-22

**Authors:** Giulia Collodel, Cinzia Signorini, Fabiola Nerucci, Laura Gambera, Francesca Iacoponi, Elena Moretti

**Affiliations:** 1 Department of Molecular and Developmental Medicine, University of Siena, Policlinico Le Scotte, Viale Bracci 14, 53100 Siena, Italy; 2 Division of Clinical Pathology, University Teaching, Hospital of Siena, Siena, Italy; 3 Fertility Center, AGI Medica, Siena, Italy; 4 Department of Food Safety, Nutrition and Veterinary Public Health, National Institute of Health, Rome, Italy

**Keywords:** Iron metabolism, Isoprostanes, Leukocytospermia, TEM, Testosterone, Varicocele

## Abstract

The evaluation of the seminal plasma plays a relevant role in the definition of male infertility and in assisted reproduction outcomes; for this reason, it would be recommended to find biochemical markers able to characterize sperm pathology. In this study, 53 infertile patients (grouped by the presence leukocytospermia, idiopathic infertility, or varicocele) and 10 fertile men were selected. Spermiogram was performed by light microscopy, and sperm ultrastructure was evaluated by transmission electron microscopy (TEM) mathematically elaborated. Testosterone (TESTO), estradiol (E2), ferritin (FERR), iron (Fe), transferrin (TRSF), triglycerides (TRG), cholesterol (CHOL), and isoprostanes (F_2_-IsoPs) were detected in seminal plasma. Sperm characteristics and biochemical components were correlated by Spearman’s rank correlation coefficient in the whole population and in each group. The levels of TESTO and E2 were positively correlated with sperm quality in particular, and E2 was correlated with fertility index expressing the number of sperm free of ultrastructural defects evaluated by TEM. On the contrary, the indices of iron metabolism (FERR, Fe, and TRSF) were positively associated with low sperm quality and sperm necrosis, particularly in leukocytospermia and varicocele groups, pathologies in which an inflammatory status and oxidative stress condition are present. The study of the seminal plasma composition deserves attention because the levels of the various components seem to be associated with specific reproductive pathologies.

## Introduction

Seminal plasma is the protein-rich fluid part of the semen and it plays an important role in sperm metabolism as well as sperm function. The analyses of seminal enzyme activities and the concentrations of biochemical elements have been performed in men and animals [1–4] in order to establish the functions of the different components, because some of them are still obscure.

The evaluation of seminal components may be used to characterized sperm quality and pathological conditions related to male infertility [5]. Many molecules are involved in the pathophysiology of spermatozoa. Iron is an essential trace nutrient that plays an important role in general health and fertility; however, it is highly toxic if accumulated in large quantities. The excess or the deficiency may lead to defective spermatogenesis and to fertility impairment [6]. About this topic, Hashemi et al. [7] reported a negative impact of iron on human sperm motility.

Transferrin is reported to be an iron-binding protein that regulates free iron levels in biological fluid in the body [8]. In the past, Barthelemy et al. [9] proposed that the seminal fluid transferrin, that correlated with sperm concentration, could be considered as a direct index of the functional state of Sertoli cells and seminiferous tubules. Recently, it has been found that transferrin could markedly extend sperm longevity in vitro [10].

Ferritin is an intracellular protein that stores iron in the atoxic form, and it is found in the extracellular environment as a result of cellular synthesis and secretion. Seminal ferritin is found in abundance in the seminal plasma, and Sertoli cells produce approximately 70% of its contents [11].

The investigation of other different semen biochemical components may explain their role in seminal pathologies. Recently, Murgia et al. [12] indicated that seminal fluid metabolome may represent a marker for oligozoospermia. Moreover, the identification of the lipids most closely related to the reproductive success may be of great interest to the scientific community. Cholesterol is a component of the mammalian plasma membrane and its redistribution and depletion from the sperm membrane are the key parts of the spermatozoon’s preparation for fertilization [13].

Many hormones are also found among the constituents of seminal plasma. When compared with blood serum levels, concentrations of testosterone in seminal plasma are lower by almost one order of magnitude, while estradiol is in some instances higher [14]. Other authors [15, 16] observed that the seminal balance between estradiol and testosterone provides a valuable biological indicator for predicting the spermatogenic state.

Our previous paper reported that increased albumin, total proteins, ferritin levels, and γ-glutamyl transferase and creatine kinase activities concomitant with decreased amounts of alkaline phosphatase and folic acid were associated with reduced sperm parameters. These results suggested their possible involvement in male infertility [17]. In humans, many pathological conditions as varicocele, urogenital infections, leukocytospermia, and inflammation influence sperm quality as well as seminal compositions. Oxidative stress in seminal fluid may be the common denominator responsible of male infertility associated with these diseases; the increased presence of reactive oxygen species (ROS) affects seminal components and it causes alterations in sperm lipids, proteins, and DNA with consequent sperm impairment [18].

Changes in the concentrations of the trace elements in human seminal plasma may be related to sperm quality since they are involved in the maintenance of the pro-/antioxidative balance in ejaculate [19]. In the presence of oxidative stress, a non-enzymatic oxidative damage in sperm plasma membranes, particularly rich in polyunsaturated fatty acid, may generate prostaglandin-like end-products known as isoprostanes (IsoPs). Seminal F_2_-isoprostanes (F_2_-IsoPs), a specific class of IsoPs, have been indicated as a marker of sperm immaturity in semen of infertile patients with varicocele [20].

To clarify the role of specific biochemical semen components in sperm quality, we have investigated the levels of cholesterol (CHOL), estradiol (E2), testosterone (TESTO), iron (Fe), ferritin (FERR), transferrin (TRSF), and triglycerides (TRG) in seminal fluid of fertile and infertile men grouped according to their reproductive pathologies. Isoprostane (F_2_-IsoP) amount was also dosed. Semen analysis was performed by light and transmission electron microscopy (TEM) mathematically elaborated. As a result, all the variables were correlated and compared.

## Materials and Methods

### Patients

In this research, from January 2018 through December 2018, we selected 53 Italian infertile patients (aged 28–39) attending AGI Medica, Fertility Center lab for semen analysis. Infertile patients did not obtain pregnancy after 2 years of unprotected sexual intercourses; the female factor was not present.

The infertile patients were grouped as follows: 16 idiopathic infertile patients, 19 patients with varicocele, and 18 patients with abacterial leukocytospermia. Leukocytospermia has been defined as reported in WHO guidelines [21]. The varicocele group was composed by patients who underwent both physical examination and scrotal color Doppler ultrasonography analysis carried out in laboratory other than ours. Patients with subclinical and grade 1 varicocele were not included in the study.

Inclusion selection criteria were non-azoospermic men, normal karyotype, BMI < 25 kg/m^2^, normal serum hormonal levels (follicle-stimulating hormone, luteinizing hormone, testosterone, prolactin), and absence of genitourinary infections. We did not include carriers of chromosome Y microdeletions and patients showing germ cells in their ejaculates. Patients with chronic diseases and receiving radiotherapy, chemotherapy, and medication were excluded. Selected men did not take an oral antioxidant supplement for at least 4 months before the study. Men with a history of recreational drug use and alcohol consumption were not selected.

Only individuals with moderate and heavy smoking habit (> 10 cigarettes/day) were excluded [22].

The fertile group was composed of 10 individuals (aged 26–40) which fathered at least one child in the last 3 years. The fertile subjects were not affected by infections and/or anatomical problems.

All selected men provided an informed written consent before the inclusion on this research where the aims of the study were accurately described.

### Semen Analysis

Semen analysis was performed according to WHO guidelines [21]. Briefly, specimens were collected by masturbation after 3–5 days of sexual abstinence and analyzed after liquefaction for 30 min at 37 °C. Conventional semen parameters were determined: volume, pH, sperm concentration, motility, and vitality. The sperm morphology was assessed by the stain-coated testsimplets slides (Origio, Italy). Peroxidase stain was used to identify the leukocytes in semen samples. A leukocyte concentration ≥ 1 million cell/ml was considered abnormal [21].

After semen evaluation, all the samples were divided into three aliquots to be used for different analyses.

An aliquot was processed for transmission electron microscopy, and the seminal volume was doubled with cold Karnovsky fixative and maintained at 4 °C for 2 h. Then samples were centrifuged at 200*g* for 15 min, the supernatant was discarded, and sperm cells were washed in 0.1 mol/l cacodylate buffer (pH 7.2) for 12 h, postfixed in 1% buffered osmium tetroxide for 1 h at 4 °C, and it was washed again in 0.1 mol/l cacodylate buffer; the samples were dehydrated in a graded ethanol series and embedded in Epon Araldite. Ultra-thin sections were cut with a Supernova ultramicrotome (Reickert Jung, Vienna, Austria), mounted on copper grids, stained with uranyl acetate and lead citrate, and at least observed and photographed with a Philips CM12 (Philips Scientifics, Eindhoven, The Netherlands) at the CE.M.E, CNR (Via Madonna del Piano, 10, 50019 Sesto Fiorentino, Italy). Three hundred sperm sections were analyzed from each sample. The TEM data was elaborated using a mathematical formula [23] which provides numerical scores such as fertility index (number of sperm free of structural defects in a semen sample) and percentage of sperm pathologies such as immaturity, apoptosis, and necrosis [24], defined by distinctive ultrastructural characteristics. Sperm immaturity include the presence of cytoplasmic droplets, altered acrosomes, roundish or elliptical nuclei with uncondensed chromatin. Marginated chromatin, translucent vacuoles included in cytoplasmic residues, swollen and badly assembled mitochondria are ultrastructural indicators of apoptosis. Sperm with reacted or absent acrosome, misshaped nuclei with disrupted chromatin, broken plasma membrane, and poor axonemal and periaxonemal cytoskeletal structures are affected by necrosis.

### Clinical Biochemistry Determinations

A semen aliquot of each ejaculate was centrifuged at 12,000*g* to obtain a sample free of cells as reported by Feng et al. [1], and the seminal plasma was collected by micropipette and stored in 2-ml cryotubes at − 80 °C until use, then thawed at room temperature. Seminal plasma samples (1 ml at room temperature) were tested using a COBAS 8000 modular analyzer (Roche Diagnostics, Mannheim, GmbH, Germany) by means of two analytical modules: C702, the high-throughput clinical chemistry module, and E602, the immunoassay module.

We measured the following parameters in seminal plasma: iron (Fe, μg/dl), triglycerides (TRG, mg/dl), cholesterol (CHOL, mg/dl), transferrin (TRSF, mg/dl), estradiol (E2, pg/μl), ferritin (FERR, ng/ml), testosterone (TESTO, ng/ml). For the analytes measured in module C702 (Fe, TRG, CHOL, TRSF), COBAS 8000 calibration was done with the human lyophilized serum Calibrator C.f.a.s. (Roche Diagnostics, Mannheim, GmbH, Germany). The C.f.a.s. (calibrator for automated systems) is a universal calibrator for adjusting most photometric methods.

Human lyophilized serum PreciControl ClinChem level 1 was used as normal control and PreciControl ClinChem level 2 was used as pathologic control (Roche Diagnostics, Mannheim, GmbH, Germany).

For the analytes measured in module E602 (FERR, E2, TESTO), we used specific calibrator for each analyte (Roche Diagnostics, Mannheim, GmbH Germany). PreciControl Varia levels 1 and 2 (Roche 470 Diagnostics, Mannheim, GmbH, Germany), PreciControl Universal levels 1 and 2 (Roche Diagnostics, Mannheim, GmbH, Germany), and PreciControl Tumor Marker levels 1 and 2 (Roche 470 Diagnostics, Mannheim, GmbH, Germany) were used as normal and pathologic controls respectively.

### F_2_-Isoprostane Determination

**An aliquot of each semen sample was centrifuged at 200 g for 15 min, then the pellet was discarded. Seminal sample, devoid of cells, was supplemented with butylated hydroxytoluene (BHT, final concentration 100** μM**) and was stored at − 80 °C until use.**

F2-isoprostanes (F_2_-IsoPs) are originally formed in situ, when the fatty acid precursor is esterified in phospholipids and subsequently released in biological fluids in a free-form through a process likely mediated by a phospholipase A_2_ (PLA_2_) activity(ies) [25]. Here, total (free plus esterified) F_2_-IsoPs were analyzed by gas chromatography/negative-ion chemical ionization-tandem mass spectrometry (GC/NICI-MS/MS). After collection, as previously reported, BHT was added in order to avoid unspecific oxidation processes. For each sample, basic hydrolysis was carried out by incubation at 45 °C for 45 min in the presence of 1 N KOH; at the end of the incubation, HCl 1 N was added to stabilize a value of pH 3.0. At this stage, in each sample, tetradeuterated derivative of prostaglandin F_2α_ (PGF_2α_-d4) (500 pg) was added as an internal standard, and solid-phase extractions were consecutively carried out on an octadecylsilane (C18) cartridge and an aminopropyl (NH_2_) cartridge. The NH_2_ final eluate was derivatized to convert the carboxyl group of F_2_-IsoPs into pentafluorobenzyl ester and the hydroxyl group into trimethylsilyl ethers [26]. For 8-iso-PGF_2α_, also referred as 15-F_2_t-IsoP, one of the most represented isomers of F_2_-IsoPs, the product ion at *m*/*z* 299, derived from the [M−181]^−^ precursor ion (*m*/*z* 569), was detected and referred to the internal standard PGF_2α_-d4 (*m*/*z* 573 → *m*/*z* 303) [27].

### Statistical Analysis

Data were reported as medians and interquartile range (IQR 75°–25° centile). The comparisons between groups (fertile, idiopathic infertility, varicocele, leukocytospermia) were evaluated by Kruskal-Wallis test followed, only for significant cases, by Dunn’s post hoc test for multiple comparisons. Spearman rho (*R*) coefficient was calculated to measure the correlation between variables for all enrolled patients and separately for each group. A *P* value < 0.05 (two-tailed) was considered statistically significant. All analyses were performed by IBM SPSS Statistics Software v.25.

## Results

**Fig. 1:**
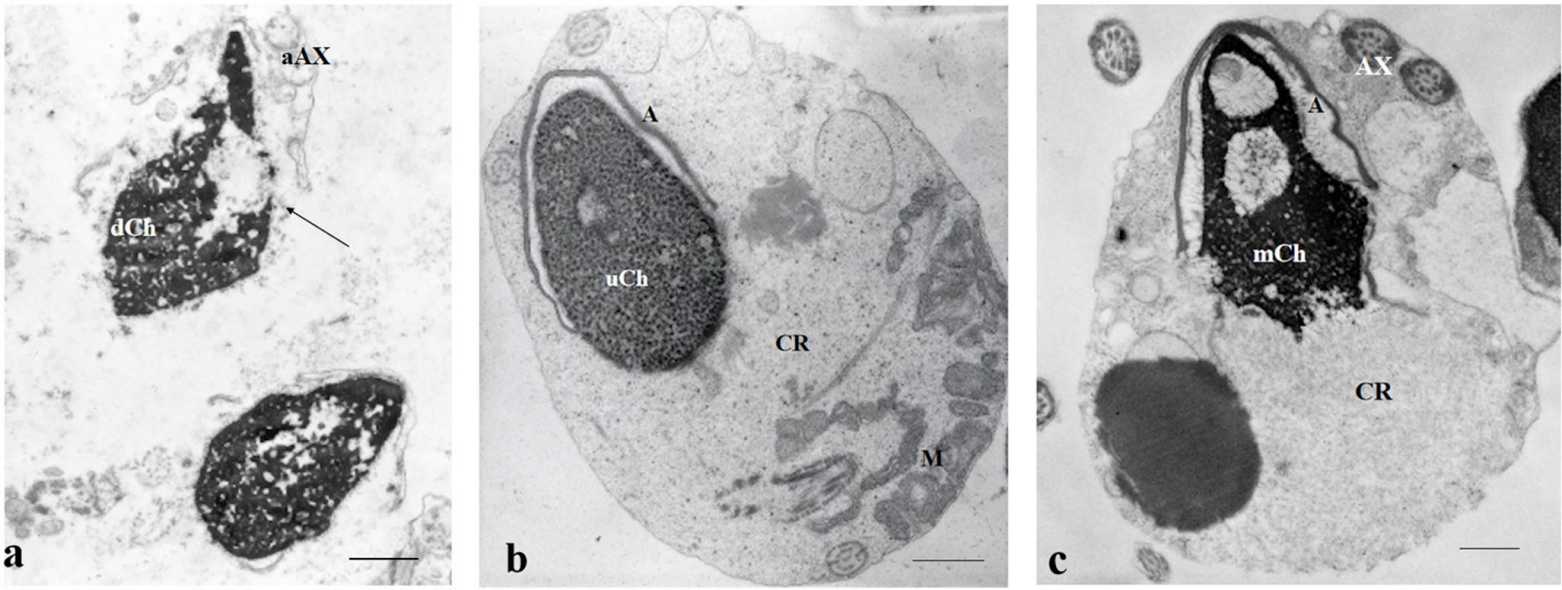
TEM micrographs of longitudinal sections of spermatozoa. In **a**, two necrotic spermatozoa are shown, the chromatin (dCh) appears disrupted, the plasma membranes are broken (arrow), and the axonemal components are absent (aAx). An immature sperm is represented in **b**, the chromatin is uncondensed (uCh), the acrosome (A) reduced and far from the nucleus, the tail is coiled into a cytoplasmic residue (CR), and mitochondria (M) are disassembled. An apoptotic sperm is shown in **c**, the chromatin is marginated (mCh), the tail is coiled but the axonemal components are well assembled (Ax); acrosome (A); and cytoplasmic residue (CR). **a**–**c** Bar, 1 μm

Semen characteristics of the infertile patients with leukocytospermia, idiopathic infertility, varicocele, and fertile group are reported in Table 1. Idiopathic infertility group significantly differed in almost all evaluated sperm characteristics from fertile men. Patients with leukocytospermia showed a significant decrease in sperm motility, normal sperm morphology, and vitality with respect to fertile patients, and higher percentage of necrosis (Fig. 1a) compared to that observed in fertile and varicocele groups (*P* < .001). Finally, in varicocele patients, immaturity percentage was significantly elevated (*P* < .001) with respect to that detected in fertile, idiopathic infertility, and leukocytospermia groups (Table 1).

**Table 1:** Medians and interquartile ranges (IQR 75°–25° centile) of semen parameters evaluated by light microscopy, sperm apoptosis, necrosis, immaturity, and fertility index evaluated by transmission electron microscopy in semen samples of 63 men divided into 4 groups (leukocytospermia, L; idiopathic infertility, I; varicocele, V; fertile men, F) according to their clinical condition. Statistics are also reported: *P < .05, **P < .01, ***P < .001

Variables	Diagnosis				Statistics	
	Leukocytospermia (L no. 18)	Idiopathic infertility (I no. 16)	Varicocele (V no. 19)	Fertile men (F no. 10)	Kruskal-Wallis (*P* value)	Multiple comparisons
Volume (ml)	3.25 (2.13)	3.75 (1.50)	4.00 (0.50)	4.25 (1.13)	0.068	
Sperm ml × 10^6^	40.50 (68.35)	6.50 (28.56)	38.50 (36.38)	118.50 (128.06)	***	F vs I
Progressive motility %	25.50 (15.50)	26.50 (19.75)	25.00 (24.00)	48.00 (30.25)	*	F vs I; F vs L
normal morphology %	9.00 (3.25)	7.50 (4.75)	10.00 (7.00)	16.00 (7.75)	***	F vs I; F vs L
Vitality %	65.00 (30.50)	66.50 (28.75)	75.00 (21.00)	85.00 (12.75)	**	F vs I; F vs L
Apoptosis %	6.48 (3.42)	9.11 (6.83)	4.13 (1.65)	4.13 (0.86)	***	F vs I; I vs V
Necrosis %	44.00 (19.95)	37.74 (10.72)	34.24 (19.65)	30.05 (11.11)	***	F vs L; L vs V
Immaturity %	50.65 (23.04)	53.89 (13.15)	75.09 (10.89)	47.20 (8.26)	***	F vs V; I vs V; L vs V
Fertility index	266,546 (785,647)	6811 (420,986)	287,471 (1,497,605)	4,025,583 (5,444,460)	***	F vs I; F vs L; F vs V

The medians and interquartile ranges of seminal levels of F_2_-IsoPs, CHOL, E2, TESTO, Fe, FERR, TRSF, and TRIG are shown in Table 2.

**Table 2:** Medians and interquartile ranges (IQR 75°–25° centile) of isoprostanes (F_2_-IsoPs), cholesterol (CHOL), estradiol (E2), testosterone (TESTO), iron (Fe), ferritin (FERR), transferrin (TRSF), and triglycerides (TRG) assayed in semen samples of 63 men divided into 3 groups according to their clinical condition (leukocytospermia, L; idiopathic infertility, I; varicocele, V; fertile men, F). Statistics are also reported: *P < .05, ***P < .001

Variables	Diagnosis				Statistics	
	Leukocytospermia (L no. 18)	Idiopathic infertility (I no. 16)	Varicocele (V no. 19)	Fertile men (F no. 10)	Kruskal-Wallis (*P* value)	Multi-comparison test
F_2_-IsoPs (ng/ml)	27.22 (31.50)	7.03 (32.63)	76.16 (55.46)	8.56 (17.42)	***	I vs V; F vs V; L vs V
CHOL (mg/dl)	22.00 (14.75)	19.00 (10.00)	17.00 (9.00)	19.00 (13.75)	0.326	
E2 (pg/μl)	45.83 (27.77)	55.63 (16.57)	58.93 (29.24)	47.09 (19.36)	0.154	
TESTO (ng/ml)	0.94 (0.37)	1.04 (1.33)	1.10 (2.03)	1.26 (1.57)	0.264	
Fe (μg/dl)	23.00 (16.50)	13.00 (8.25)	15.00 (10.00)	14.50 (11.00)	*	I vs L
FERR (ng/ml)	337.2 (179.4)	233.9 (91.3)	238.2 (77.3)	158.4 (75.8)	***	L vs F; L vs V; L vs I
TRSF (mg/dl)	1.50 (7.00)	0.50 (1.00)	3.64 (3.00)	0.50 (3.00)	0.117	
TRG (mg/dl)	99.50 (181.75)	7.00 (59.25)	2.00 (126.00)	56.50 (164.50)	0.053	

F_2_-IsoP level, a reliable indicator of oxidative stress, was significantly increased in varicocele group compared to leukocytospermia, idiopathic infertility, and fertile groups (*P* < .001). The values of CHOL, E2, TESTO, TRSF, and TRG did not show differences in the considered groups. In leukocytospermia group, the concentration of FERR was significantly increased than that observed in fertile, varicocele, and idiopathic infertility groups (*P* < .001) and the Fe level was higher than that detected in idiopathic infertility group (*P* < .05).

In order to understand the possible correlations among the studied variables, we used the Spearman rank correlation coefficient considering the whole population of interest (Table 3). Regarding biochemical components, F_2_-IsoP levels correlated positively with sperm immaturity (Fig. 1b; *P* < .01). Positive correlations between the TRSF (*P* < .01) and TRG (*P* < .05) levels and sperm concentration were observed. FERR showed negative correlations with sperm morphology, vitality, and fertility index (*P* < .05) and positive correlations with sperm necrosis (*P* < .01) and Fe (*P* < .05); Fe compound also positively correlated with sperm necrosis (*P* < .05). Interestingly, TESTO displayed positive correlations with the sperm vitality (*P* < .05) and E2 level (*P* < .01) and negative correlations with TRG (*P* < .01) and sperm apoptosis (Fig. 1c; *P* < .05).

**Table 3:** Correlations (rho Spearman’s coefficient) between all considered variables in 63 individuals

	Sperm/ml × 10^6^	Motility %	Normal morphology %	Vitality%	A %	N %	I %	FI	E2	FERR	Fe	TESTO	TRG	TRSF
Sperm/ml × 10^6^	1													
Motility %	.400**	1												
Normal morphology %	.677**	.753**	1											
Vitality%	.271*	.639**	.645**	1										
A %	NS	NS	NS	− .359**	1									
N %	− .299*	− .250*	− .462**	− .454**	.356**	1								
I %	NS	− .264*	NS	NS	NS	NS	1							
FI	.697**	.522**	.642**	.392**	NS	− .457**	− .263*	1						
E2 (pg/μl)	NS	NS	NS	NS	NS	NS	.303*	NS	1					
FERR (ng/ml)	NS	NS	− .259*	− .285*	NS	.562**	NS	− .253*	NS	1				
Fe (μg/dl)	NS	NS	NS	NS	NS	.253*	NS	NS	NS	.314*	1			
TESTO (ng/ml)	NS	NS	NS	.272*	− .254*	NS	NS	NS	.581**	NS	NS	1		
TRG (mg/dl)	.248*	NS	NS	NS	NS	NS	NS	NS	NS	NS	NS	− .357**	1	
TRSF (mg/dl)	.326**	NS	NS	NS	NS	NS	NS	NS	NS	NS	.306*	NS	NS	1
F_2_-IsoPs (ng/ml)	NS	NS	NS	NS	NS	NS	.640**	NS	NS	NS	NS	NS	NS	NS

*Sperm/ml × 10*^*6*^, number of sperm/ml; *motility %*, percentage of progressive sperm motility; *normal morphology %*, percentage of normal sperm morphology assessed with testsimplets; *vitality %*, percentage of viable sperm; *A %*, percentage of sperm apoptosis assessed by transmission electron microscopy; *N %*, percentage of sperm necrosis assessed by transmission electron microscopy; *I %*, percentage of sperm immaturity assessed by transmission electron microscopy; *FI*, fertility index, the number of sperm probably devoid of ultrastructural defects; *F*_*2*_*-IsoPs*, isoprostanes; *CHOL*, cholesterol; *E2*, estradiol; *TESTO*, testosterone; *Fe*, iron; *FERR*, ferritin; *TRSF*, transferrin; *TRG*, triglycerides, *NS*, not significant

*P < .05, **P < .01

Different correlations between biochemical indices and sperm parameters were analyzed in each studied group (Table 4, Spearman’s rank correlation coefficient).

**Table 4:** Correlations (rho Spearman’s coefficient) between all considered variables in 63 individuals grouped in fertile men (line 1), idiopathic infertile patients (line 2), leukocytospermic men (line 3), and infertile patients with varicocele (line 4)

	Sperm/ml × 10^6^	Motility %	Normal morphology %	Vitality %	A %	N %	I %	FI	E2	FERR	Fe	TESTO	TRG	TRSF	CHOL
Sperm/ml × 10^6^	1														
Motility %	NS NS .486* NS	1													
Normal morphology %	.671* NS .693** .656**	.725* .650** .705** .712**	1												
Vitality%		NS .635** .618** .534*	NS .766** NS .519*	1											
A %				NS NS − .489* − .500*	1										
N%				NS NS − .579* NS	NS NS .627** NS	1									
I %	NS − .570* NS NS	NS NS NS − .498*	− .695* NS NS − .476*				1								
FI	NS .560* .650** .706**	NS NS .605** .575**	NS NS .602** .691**			NS NS NS − .502*		1							
E2 (pg/μl)								.709* NS NS NS	1						
FERR (ng/ml)		NS NS NS − .493*				NS NS NS .487*			NS NS − .498* NS	1					
Fe (μg/dl)											1				
TESTO (ng/ml)						NS NS − .518* NS			.891** NS .752** .598**	NS NS − .586* NS		1			
TRG (mg/dl)							NS NS NS NS						1		
TRSF (mg/dl)									NS NS NS − .607**			NS NS NS − .560*	NS NS .506* NS	1	
CHOL (mg/dl)						NS NS .524* NS							.786** NS NS NS	NS NS .520* NS	1
F2-IsoPs (ng/ml)							NS NS .530* .786**								

*Sperm/ml × 10*^*6*^, number of sperm/ml; *motility %*, percentage of progressive sperm motility; *normal morphology %*, percentage of normal sperm morphology assessed with testsimplets; *vitality %*, percentage of viable sperm; *A %*, percentage of sperm apoptosis assessed by transmission electron microscopy; *N%*, percentage of sperm necrosis assessed by transmission electron microscopy; *I %*, percentage of sperm immaturity assessed by transmission electron microscopy; *FI*, fertility index, the number of sperm probably devoid of ultrastructural defects; *F*_*2*_*-IsoPs*, isoprostanes; *CHOL*, cholesterol; *E2*, estradiol; *TESTO*, testosterone; *Fe*, iron; *FERR*, ferritin; *TRSF*, transferrin; *TRG*, triglycerides; *NS*, not significant

*P < .05, **P < .01

In fertile men, E2 positively correlated with fertility index (*P* < .05) and TESTO (*P* < .01); CHOL positively correlated with TRG (*P* < .001).

In idiopathic infertility group, we did not observe significant correlations between seminal plasma components and sperm characteristics.

In leukocytospermia group, E2 positively correlated with TESTO (*P* < .01) and TESTO negatively correlated with sperm necrosis (*P* < .05); both TESTO and E2 negatively correlated with FERR (*P* < .05). TRSF had positive correlations with CHOL and TRG (both *P* < .01). Sperm immaturity positively correlated with F_2_-IsoPs (*P* < .05). Sperm necrosis is positively correlated with CHOL (*P* < .05).

In infertile patients affected by varicocele, E2 showed positive correlation with TESTO (*P* < .01), and both E2 and TESTO negatively correlated with TRSF (respectively *P* < .01; *P* < .05). FERR displayed a positive correlation with sperm necrosis (*P* < .05) and negative correlation with sperm motility (*P* < .05). Finally, F_2_-IsoPs had a positive relationship with sperm immaturity (*P* < .01).

## Discussion

The evaluation of the seminal plasma composition may play a relevant role in the definition of male infertility and in assisted reproduction outcomes [28]; for this reason, it would be recommended to find biochemical markers able to characterize sperm pathology [29]. Our and other groups have stressed on the importance of the research of seminal indicators in the different male infertility conditions [30–33]. In our recent paper, we suggested that some seminal biochemical components may be associated with pathological conditions of human semen. Patients with sperm vitality ≤ 5th percentile showed increased albumin concentration and creatine kinase activity. The presence of germ cells in semen was associated with high values of FERR; the alkaline phosphatase activity and folic acid were decreased in hyperviscous semen samples [17]. There are several studies focused on the proteomic composition of human seminal plasma, which may be linked to specific pathways of infertility [3, 34]. Mirnamniha et al. [35] reported that the deficiency of trace elements such as calcium copper, manganese, magnesium, zinc, and selenium is associated with impaired spermatogenesis, altered levels of sex hormones, seminal oxidative stress, inflammation, and apoptosis. Recently, Murgia et al. [12] detected altered levels of fructose, myo-inositol, aspartate, and choline in oligozoospermic men.

In this paper, the data regarding the analyzed variables suggested some intriguing hypotheses. The level of TESTO did not differ in the examined groups but it was strongly positively correlated with sperm quality and with E2; in turn, E2 level positively correlated with fertility index expressing the number of sperm free of ultrastructural defects evaluated by TEM. These results confirmed that these hormones have a relevant positive influence in sperm physiology and the seminal balance between estrogenic and androgenic actions is essential for maintaining spermatogenesis and male fertility [15, 16, 36]. In agreement with our data, Thanaboonyawat et al. [37] demonstrated that in vitro supplementation of TESTO determined a significant retardation in the normal reduction of sperm motility over time. Moreover, the effects of E2 on human sperm have been associated with an increased motility, oocyte penetration, longevity, oxygen intake, and metabolization of exogenous substrates [38–40]. The correlation between these hormones is justified by the fact that in adult human and animal testis, TESTO is converted to E2 by aromatase (CYP19A1), the ubiquitous NADPH cytochrome P450 reductase enzyme principally found in spermatids and mature sperm in seminiferous tubules and Leydig cells [41]. A further confirmation that TESTO is a positive marker of semen quality is suggested by its negative correlation with sperm necrosis.

On the contrary, the indices of iron metabolism (FERR, Fe, and TRSF) were positively associated with low sperm quality and negatively with TESTO and E2 levels, particularly in leukocytospermia and varicocele groups, pathologies in which an inflammatory status and oxidative stress condition are generally present [42].

The relationship among TESTO, FERR, and inflammation is well known in the literature [43–46] even if the determinations are performed in blood serum. In our study, they were detected in human seminal plasma and these associations were confirmed suggesting the involvement of cytokines [43] and ROS [47] also in the seminal fluid from patients with varicocele or leukocytospermia. It is known that an increased number of leukocytes are positively associated with altered sperm characteristics, sperm necrosis, intracellular ROS [30, 48, 49], and level of Fe in the seminal plasma of men with different fertility potentials [50]. Overload of Fe may cause disturbances to spermatogenesis as well as to crucial sperm cell structures accompanied by oxidative stress and cell death [6, 50].

FERR has been localized in human Sertoli and Leydig cells [51, 52] and an increased level of FERR was detected in human semen concomitantly to the presence of germ cells [17]. Seminal fluid TRSF values were proposed as a reliable clinical index for determining the functional status of Sertoli cells or seminiferous tubules [9]. In mice, TRSF overexpression had negative effects on testicular function and TRSF level required strict regulation in the testis [53]. In our paper, the negative correlation between TRSF and TESTO and E2 in varicocele patients may suggest a role of TRSF as an index of testis malfunctioning since it is well known that varicocele can alter Sertoli cell function and consequently influence the TSRF level.

This study confirmed the close relationship between increased levels of F_2_-IsoP varicocele and sperm immaturity observed also in previous studies [20, 54]; since these studies included different patient populations, the semen level of F_2_-IsoPs can be considered a semen biochemical marker of sperm immaturity and varicocele.

Noteworthy is the behavior of CHOL and TRG that in the first analysis seems to be irrelevant, either as marker of pathologies either in correlations performed in the whole studied population. When the correlations between variables were carried out in each group separately, we observed that in the case of leukocytospermia, CHOL and TRG positively correlated with sperm necrosis. Previous studies suggested that elevated lipid levels in seminal plasma might have adverse effect on sperm quality [55], and seminal plasma TRG and total CHOL levels had a positive correlation with sperm DNA fragmentation [56]. Also increased lipid serum levels play a negative influence on sperm quality [57].

Patients with idiopathic infertility showed an altered sperm quality as the other infertile groups; however, they did not show correlations between semen parameters and seminal plasma markers highlighted for leukocytospermia and varicocele groups. This observation could be explained by the intrinsic meaning of idiopathic infertility since it is difficult to group patients with homogeneous characteristics. Indeed, in semen of many men who are currently diagnosed as having unexplained male infertility, oxidative stress was detected, but other genetic, epigenetic, or environmental unknown causes could be supposed [58, 59].

## Conclusions

In conclusion, infertile men with varicocele, leukocytospermia, and idiopathic infertility differed in seminal fluid composition. TESTO and E2 levels appeared to be necessary for a regular spermatogenesis and play a role in sperm quality; FERR, Fe, and TRSF appeared to be linked to inflammatory conditions and sperm necrosis as well as F_2_-IsoPs to sperm immaturity. We are aware that the number of cases is low and should be extended in a future study; however, these preliminary data indicated that the seminal plasma composition deserves attention because the levels of the various components seem to be associated with specific reproductive pathologies.

The knowledge of the role of seminal molecules in male infertility will help in the development of targeted treatments.

## Data Availability

The datasets used and/or analyzed during the current study are available from the corresponding author on reasonable request.
